# Calculation of narrower confidence intervals for tree mortality rates when we know nothing but the location of the death/survival events

**DOI:** 10.1002/ece3.5495

**Published:** 2019-07-30

**Authors:** Gabriel Arellano

**Affiliations:** ^1^ Ecology and Evolutionary Biology University of Michigan Ann Arbor MI USA; ^2^ ForestGEO Smithsonian Tropical Research Institute Washington DC USA

**Keywords:** Bernoulli trial, binomial distribution, confidence intervals, demography, ForestGEO, Lambir Hills National Park, Poisson binomial distribution, spatial aggregation, tropical forest dynamics

## Abstract

1. Many ecological applications, like the study of mortality rates, require the estimation of proportions and confidence intervals for them. The traditional way of doing this applies the binomial distribution, which describes the outcome of a series of Bernoulli trials. This distribution assumes that observations are independent and the probability of success is the same for all the individual observations. Both assumptions are obviously false in many cases.
2. I show how to apply bootstrap and the Poisson binomial distribution (a generalization of the binomial distribution) to the estimation of proportions. Any information at the individual level would result in better (narrower) confidence intervals around the estimation of proportions. As a case study, I applied this method to the calculation of mortality rates in a forest plot of tropical trees in Lambir Hills National Park, Malaysia.
3. I calculated central estimates and 95% confidence intervals for species‐level mortality rates for 1,007 tree species. I used a very simple model of spatial dependence in survival to estimate individual‐level risk of mortality. The results obtained by accounting for heterogeneity in individual‐level risk of mortality were comparable to those obtained with the binomial distribution in terms of central estimates, but the precision increased in virtually all cases, with an average reduction in the width of the confidence interval of ~20%.
4. Spatial information allows the estimation of individual‐level probabilities of survival, and this increases the precision in the estimates of mortality rates. The general method described here, with modifications, could be applied to reduce uncertainty in the estimation of proportions related to any spatially structured phenomenon with two possible outcomes. More sophisticated approaches can yield better estimates of individual‐level mortality and thus narrower confidence intervals.

## 1 INTRODUCTION

One of the most fundamental problems in statistics is the estimation of proportions and the uncertainty around those estimates (Brown, Cai, & Dasgupta, 2001). Existing approaches are almost exclusively based on the binomial distribution. This distribution describes the probability of observing a given number of successes, *k*, in a series of *n* independent Bernoulli trials (any experiment with only two possible outcomes: failure = 0, success = 1), when the probability of success, *p*, is constant. The binomial distribution and the different methods of estimating confidence intervals around the estimate of *p* have been applied in every area requiring statistics. In many cases, the independence of trials or events has been assumed when it is obviously not true, resulting in overly conservative estimates of proportions. Here, I describe a method to take advantage of internal heterogeneity and/or lack of independence between observations to increase precision in the estimation of proportions. I apply the method to the calculation of mortality rates using repeated censuses (Condit, Hubbell, & Foster, 1995; Kohyama, Kohyama, & Sheil, 2018; Lewis et al., 2004; Sheil, Burslem, & Alder, 1995). In the particular case of trees (long‐lived organisms), the scarcity of death observations has resulted in great uncertainty in the estimation of mortality rates. Being this a crucial aspect of forest functioning, improvements in the estimation of mortality rates, including narrower confidence intervals, should have important implications in our understanding and prediction of forest systems (McDowell et al., 2018; McMahon, Arellano, & Davies, 2019).

**Figure 1:**
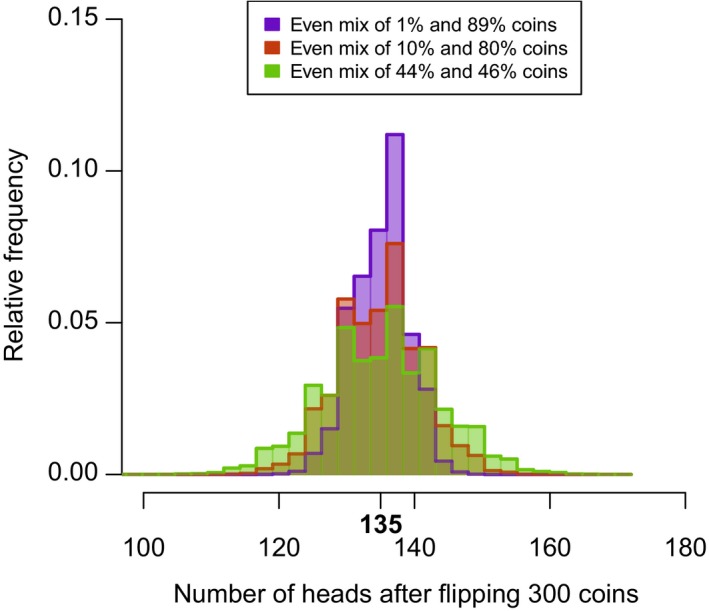
Experiment involving three mixes of 300 coins each, with varying heterogeneity in coin biases. In all cases, the expected number of heads is the same (*k* = 135). However, the variability in the outcome of the experiment is lower when the heterogeneity present in the mix of coins is greater

Let us start with a coin tossing experiment involving many coins. Intuitively, the heterogeneity in *p_i_* (the coin‐level probability of heads) should reduce the uncertainty about the outcome of *n* coin tosses. For example, if we knew that all coins were either two‐headed or two‐tailed, there would be little uncertainty about the outcome. In contrast, if all coins were fair (50% chance of head and 50% chance of tail) the uncertainty will be greater. This is what one can observe when tossing mixes of coins with different levels of bias: the greater the heterogeneity in coin‐level *p_i_*, the lower the variability in the outcome (Figure 1). Of course, this is of no practical application if we do not know anything about the individual‐level probability of success, *p*_1_, *p*_2_, …, *p_n_*. How to estimate such vector of probabilities is a domain‐specific problem to a large degree. However, the dependence between observations is in and of itself a general (not domain‐specific) source of very valuable information about *p*_1_, *p*_2_, …, *p_n_*.

**Figure 2:**
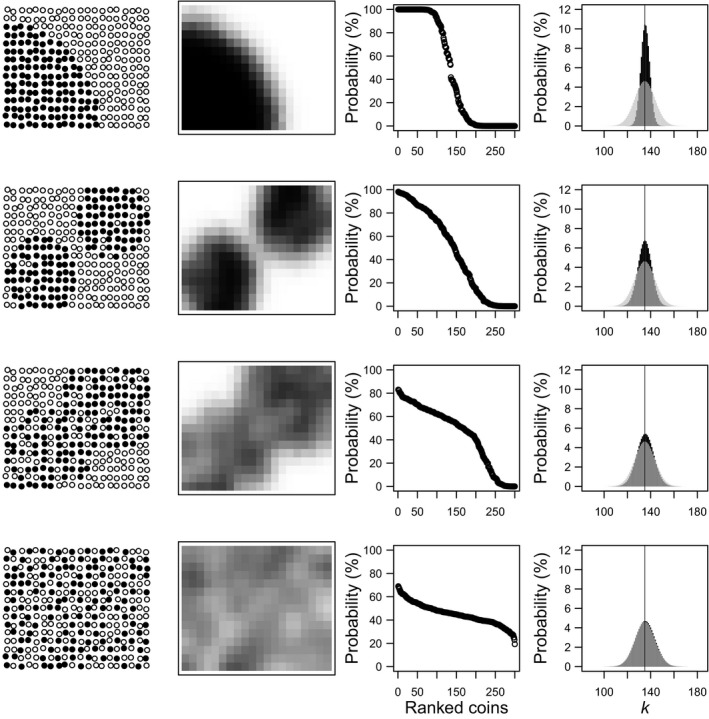
Experiment involving tossing 300 coins in a table. The table is 20 × 15 in size and coins are arranged in a 1 × 1 grid (arbitrary units). First column: the outcome of the experiment, each coin tossed results in either head (black) or tail (white). We know nothing but the outcome of each toss and its location. It is clear that there are spatial processes at play: either the coins were arranged according to their inherent biases, or some hidden process is biasing the result of each coin toss (e.g., a magnetic device under the table). Second column: the estimated map of the “probability of head” using Gaussian kernel estimation (*σ* = 1.25 in all cases). This estimation is completely agnostic regarding the underlying mechanism. Third column: the 300 coins ranked by the estimated coin‐level probability of heads. That is, a nonspatial representation of the estimated vector of probabilities of resulting in head (${\hat{p}}_{1},\;{\hat{p}}_{2},\;\ldots ,\;{\hat{p}}_{300}$). Fourth column: comparison between the expectations by the binomial distribution (gray bars) and the Poisson binomial distribution (black bars). Bars represent the probability of observing a given number of heads, *P*(*k*), according to both distributions. Both differ when there is strong spatial pattern and tend to match when there is no spatial pattern (i.e., random distribution of heads/tails, or homogeneous underlying probability of heads)

Many fear statistical dependence. In fact, it is seen by most practitioners as something negative, absolutely bad, something that decreases the effective sample size, and systematically leads us to wrong or uncertain conclusions. This is, in the best case, a biased perception. It is true that models that assume independence between observations will fail when this assumption is not met. However, if we think strictly about data (not models) it is clear that statistical dependence reduces uncertainty about the outcome, as long as we can measure or estimate it. For example, light levels at the one‐minute scale are so strongly autocorrelated during the day that there is almost no uncertainty whatsoever about what to expect. That is why solar eclipses are such powerful subjective experiences for visual organisms, including *Homo sapiens* (personal observation). The same reasoning applies to other types of dependence, like spatial dependence. If we are tossing many coins arranged in a table and we see spatial aggregation in the resulting heads/tails, we can (should) suspect that other processes than the inherent coin biases are at play. Perhaps the coins were organized prior to the experiment by someone else according to some criterion, or there is a hidden magnetic device under the table influencing the results, etc. It does not matter what is the nature of the process: the spatial aggregation in the results is enough to infer about heterogeneity in the coin‐level probability of heads, and the expected variability in (uncertainty about) the possible outcome (Figure 2).

Dependence between observations, including spatial dependence, is a frequent feature of ecological data, and the reasoning presented above applies to plant demography. We can imagine a plant survey containing two species equally abundant: one of them being a very long‐lived plant (e.g., zero deaths after one year, out of 1,000 individuals) and the other being annual (1,000 deaths after one year, out of 1,000 individuals). Although the community‐level mortality rate is 50%/year, and it is true that a randomly chosen individual has a 50% chance of dying during a given year, the individual‐level probability of dying or surviving is actually either ~0 or ~1, and thus the possible variability in outcome is, in reality, much lower. We would not be throwing 2,000 approximately fair coins, but 2,000 strongly biased coins. Furthermore, we could generally expect this imaginary plant community to show some kind of spatial structure, perhaps by being a mosaic of monospecific patches. Even if we cannot distinguish both species (i.e., even in the absence of taxonomic information), as long as there is some kind of spatial pattern, we can estimate whether a given individual or position is more/less likely to survive than *k*/*n*. In fact, if we can draw a reasonable probability map, any spatial pattern in the sample will increase precision in mortality rates estimates for the entire system.

## 2 METHODS

### 2.1 The bootstrap paradigm of inference and the Poisson binomial distribution

Bootstrap is a general paradigm for inference based on repeated sampling. It facilitates creating confidence intervals on any statistic without requiring many assumptions (in particular, without requiring the assumption of independence of observations). Suppose we have a sample **x** = (*x*_1_, *x*_2_, …, *x_n_*) from an unknown probability distribution *F*, and that we want to estimate some property of the system *θ* = *t*(*F*). The standard approach is to use the sample **x** and then calculate some statistic *s*(**x**) on it, so our estimate of *θ* is $\hat{\theta }=s\left(x\right)$. In contrast, the bootstrap approach requires creating an “alternative world” ($\hat{F}$) and then taking samples from it, $x^{*}$, so $\hat{\theta }=t(\hat{F})=s(x^{*})$. Confidence intervals for $\hat{\theta }$ can be obtained as quantiles in its distribution after sampling many times from $\hat{F}$ (i.e., after obtaining $x_{1}^{*},\;x_{2}^{*},\;x_{3}^{*},$ etc.).

Bootstrap is a very well‐known technique, and a discussion of its implications and applications goes beyond the scope of this study. However, I want to emphasize that $\hat{F}$ does not need to be the empirical distribution of **x**. Although this is certainly the most frequent approach, one can (must) build the bootstrap world $\hat{F}$ so that it is similar to *F* in the ways that matter most. In our case, we are interested in creating $\hat{F}=\left({\hat{p}}_{1},\;{\hat{p}}_{2},\;\ldots ,\;{\hat{p}}_{n}\right)\in \left[0,1\right]$ (an estimation of the coin biases, the individual‐level risk of mortality) more than getting a series of binary data (head/tail, dead/alive) directly by sampling the observations with replacement. The latter would result in the bootstrapped confidence interval for the binomial distribution and still reflects the (conservative) assumption of constant probability of success. That is, it is equivalent to using $\hat{F}=\left({\hat{p}}_{1}={\hat{p}}_{2}=\ldots ={\hat{p}}_{n}\right)$, which does not capture the key aspect of $\hat{F}$ in which we are interested: heterogeneity.

The bootstrap approach is useful in general both to obtain central estimates and confidence intervals. There is also an analytical shortcut for the probability of *k* successes in a series of *n* Bernoulli trials with varying probability of success, which is described by the Poisson binomial distribution. The exact expression is numerically intractable in most cases, but there are some useful approximations, like this (Fernández & Williams, 2010; Hong, 2013):

(1) $$P\left(k|;n,\;p_{1},\;p_{2},\;\ldots ,\;p_{n}\right)\approx \frac{1}{n+1}\sum_{i=0}^{n}C^{-ik}\prod_{j=1}^{n}\left(1+\left(C^{i}-1\right)p_{j}\right)$$

where $C=e^{2\pi \sqrt{-1}/\left(n+1\right)}$. The *k* for which *P*(*k*|*n*, *p*_1_, *p*_2_, …, *p_n_*) is maximized is the most likely number of successes during the series of trials, $\hat{k}$. Note that the binomial distribution is just a special case of the Poisson binomial distribution when *p*_1_ = *p*_2_ = ⋯ = *p_n_* = *k*/*n*.

### 2.2 Individual‐level probability of survival in tropical trees

If we are able to get a meaningful vector of probabilities of individual survival (${\hat{p}}_{1},\;{\hat{p}}_{2},\;\ldots ,\;{\hat{p}}_{n}$), we can calculate by bootstrapping narrower confidence intervals than those obtained using the binomial distribution. The challenge is to obtain this vector of probabilities. This is, in general, a domain‐specific problem. In the case of trees, there are many ways to estimate individual‐level risk of mortality if one has individual‐level covariates like tree size, crowding, previous growth, crown damage, functional traits (e.g., Arellano, Medina, Tan, Mohamad, & Davies, 2019; Camac et al., 2018; Iida et al., 2014; Kohyama et al., 2018; Rüger, Huth, Hubbell, & Condit, 2011). These methods require in most cases detailed data, above‐average modeling skills, and, very often, substantial computational resources. Here, I use a very simple model to draw a spatial map of probabilities and apply the Poisson binomial distribution to estimate species‐level mortality rates using empirical data on tropical forest trees. The approach is simple and can be applied even when we know nothing except the location of the observations. It is certainly not the perfect approach, and possibly not the most desirable in case one has access to relevant covariates and modeling resources/skills.

#### 2.2.1 Forest data

I calculated species‐level mortality rates for all the species with at least 20 stems in the 52‐ha permanent plot at Lambir Hills National Park, Malaysian Borneo (4°12′N, 114°01′E) (Lee, Ashton, et al., 2002; Lee, Davies, et al., 2002). The forest is a lowland evergreen rainforest dominated by Dipterocarpaceae, receiving >3,000 mm of rainfall per year (Davies, Tan, LaFrankie, & Potts, 2005). The plot is on dissected terrain, ranging from 109 to 240 m above sea level. The plot includes a gradient in soil moisture and fertility. Ridges are drier and have a low‐fertility sandy loam soil, while the lower slopes and valleys are wetter and have more fertile clay soils (Tan et al., 2009). The plot was established in 1991–1992 and recensused in 1997, 2003, and 2008 following the standard protocol of the Center for Tropical Forest Science—ForestGEO network (Condit, 1998; Manokaran et al., 1990). Here, I focus on the time interval between 2003 and 2008.

#### 2.2.2 Creation of the probability map

Consider a binary variable *x* taking value dead = 0 or alive = 1, expressed in *n* points. Assume that the status of point *i* is not independent of the status of all the other points and that the distance between points plays a role. The set of observations with *x* = 1 is denoted as *U* and the set of observations with *x* = 0 is denoted as *Z*. Our goal is to map *P*(*x* = 1) in the space to assign a spatially explicit probability of success to each point in the space, which translates into a sequence of probabilities of survival for each tree: ${\hat{p}}_{1},\;{\hat{p}}_{2},\;\ldots ,\;{\hat{p}}_{n}$. The spatial dependence will be included as long as the estimated ${\hat{p}}_{i}$ of each tree is influenced, to some degree, by the neighboring trees.

There are many options to estimate a probability map. I used a simple model, with one parameter, that estimates the probability density function of *P*(*x_i_* = 1) based on points in *U* and the probability density function of *P*(*x_i_* = 0) based on points in *Z*. These densities, *D*, can be estimated using standard Gaussian kernels:

(2) $$D\left(x_{i}=1\right)=\sum_{\begin{array}{c} j\in U\\ j\neq i \end{array}}g(d_{\mathrm{ij}},\sigma )$$

(3) $$D\left(x_{i}=0\right)=\sum_{\begin{array}{c} j\in Z\\ j\neq i \end{array}}g(d_{\mathrm{ij}},\sigma )$$

where *d_ij_* is the distance between points *i* and *j*, and *g* is the Gaussian kernel centered at zero distance and standard deviation *σ*. These two densities can be thought of as relative probabilities of *x_i_* = 1 and *x_i_* = 0. We can estimate the probability of tree‐level survival as ${\hat{p}}_{i}=P\left(x_{i}=1\right)=D\left(x_{i}=1\right)/\left(D\left(x_{i}=1\right)+D\left(x_{i}=0\right)\right)$. This sequence (${\hat{p}}_{1},\;{\hat{p}}_{2},\;\ldots ,\;{\hat{p}}_{n}$) was rescaled to ensure that the most likely number of survivors matched the observed number of survivors and that no tree had ${\hat{p}}_{i}=0$ or ${\hat{p}}_{i}=1$ exactly, by substituting these numbers by 0.01% and 99.99%, respectively.

**Figure 3:**
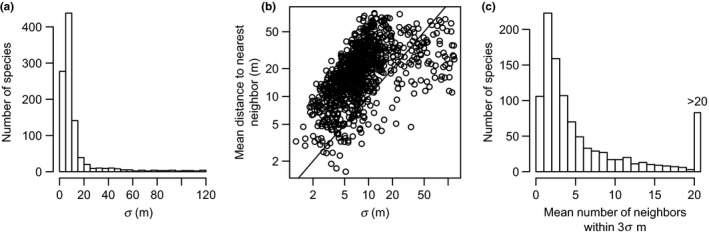
Bandwidth (*σ*) of the Gaussian kernels underlying the survival probability maps of 1,007 canopy tree species present in the Lambir 52‐ha permanent plot during the 2003–2008 period. (a) Distribution of species‐level *σ*, which were chosen as the distance of maximum aggregation of dead individuals or maximum repulsion between surviving individuals. (b) Species‐level *σ* versus the species‐level mean distance between one individual and its closest conspecific neighbor. (c) Mean number of conspecifics within 3*σ* distance to any given individual, roughly the 95th percentile of the two‐dimensional Gaussian distribution. This panel gives an approximate idea of how many individuals were used for estimation of the local or individual‐level survival rates

The selection of *σ* must be relevant considering the spatial scale of the studied phenomenon. In the case of trees, it should be similar in magnitude to the distances at which trees influence (or inform about) each other in the aspects relevant for short‐term survival (e.g., gap dynamics, Janzen‐Connell processes, aggregated mortality in certain locations because of flooding, or other localized stresses). In general, it is impossible to know about the nature of all these interacting processes when we just have the coordinates. Here, I applied an omnibus solution based on the spatial pattern alone, as measured by *L*(*r*), the Besag's transformation of Ripley's *K*‐function. This function reflects the amount of observations within distance *r* around any given observation. I compared the observed *L*(*r*) for dead or surviving individuals (*L*_dead_(*r*) or *L*_surv_(*r*)) with the observed *L*(*r*) for all the individuals together, *L*_all_(*r*), which functions as a general expectation. In particular, I calculated the relative aggregation of dead and surviving individuals at any given scale as *A*_dead_(*r*) = (*L*_dead_(*r*) − *L*_all_(*r*))/*L*_all_(*r*) and *A*_surv_(*r*) = (*L*_surv_(*r*) − *L*_all_(*r*))/*L*_all_(*r*). The distance that maximizes *A*_dead_(*r*) informs about the distance at which different processes have been killing trees during the observed period. The distance that minimizes *A*_surv_(*r*) informs about the distances at which different processes have been killing trees longer‐term, during all the years that resulted in the current configuration of the spatial distribution of a that particular species in the forest. I calculated both distances and chose the shortest as the species‐level *σ*. These *σ* values varied considerably between species but were typically <20 m (~12 m in average, Figure 3). All these calculations were based on the *Lest* function in the *spatstat* R package v. 1.54.0 (Baddeley, Rubak, & Turner, 2015), with the isometric correction and default parameters.

#### 2.2.3 Mortality rates

**Figure 4:**
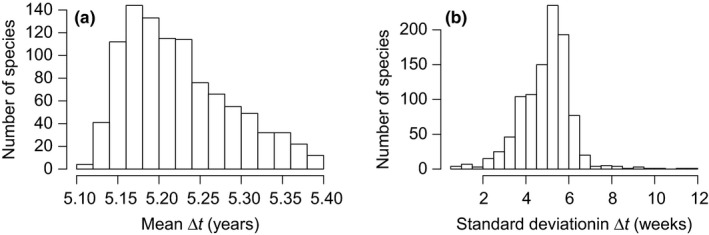
Interval length between two censuses happening in 2003 and 2008 in the 52‐ha permanent plot in Lambir (Malaysia). (a) Distribution of the mean interval length, which was incorporated into the calculation of species‐level mortality rates. (b) Distribution of the individual variability of interval length, within any given species. This information was disregarded and not included in the calculation of mortality rates, as is common practice in tropical forest ecology (Kubo, Kohyama, Potts, & Ashton, 2000)

The mortality rates for each species were calculated as (Sheil et al., 1995) $\hat{\lambda }=\left(\log n-\log\hat{k}\right)/\Delta t$, where Δ*t* was the average difference in census dates across the *n* individuals of that species, and $\hat{k}$ was the most likely number of survivors, given species‐level *n* and ${\hat{p}}_{1},\;{\hat{p}}_{2},\;\ldots ,\;{\hat{p}}_{n}$. Δ*t* was ~5.20 years in average, and the typical variation in census dates for any given species was ~5 weeks around Δ*t* (Figure 4). $\hat{k}$ was calculated according to the implementation of the Poisson binomial distribution in the *poisbinom* R package v. 1.0.1 (Olivella & Shiraito, 2017). The 95% confidence intervals for $\hat{\lambda }$ were calculated by bootstrapping values for *x* based on ${\hat{p}}_{1},\;{\hat{p}}_{2},\;\ldots ,\;{\hat{p}}_{n}$, and then calculating λ* based on k* (the number of survivors in each bootstrap sample x*). I repeated this 10,000 times and estimated confidence intervals for $\hat{\lambda }$ as the 2.5% and 97.5% quantiles in the distribution of λ*. I proceeded the same for the Poisson binomial distribution and for the binomial distribution, which is just a special case with *p*_1_ = *p*_2_ = ⋯ = *p*_n_ = *k*/*n*. Applied to the binomial distribution, this method is equivalent to obtaining confidence intervals by bootstrapping the original observations (e.g., van Breugel et al., 2011; Thomas, Kellner, Clark, & Peart, 2013).

The R code required for all the calculations, included a general wrapper for convenience, is presented in Appendix S1.

## 3 RESULTS

I calculated stem mortality rates with 95% confidence intervals for 1,007 species of trees with at least 20 stems in the Lambir plot. For other eight species, *k* = *n* or *k* = 0 and I could not estimate a realistic probability map (i.e., a map with probabilities different than 0 or 1).

**Figure 5:**
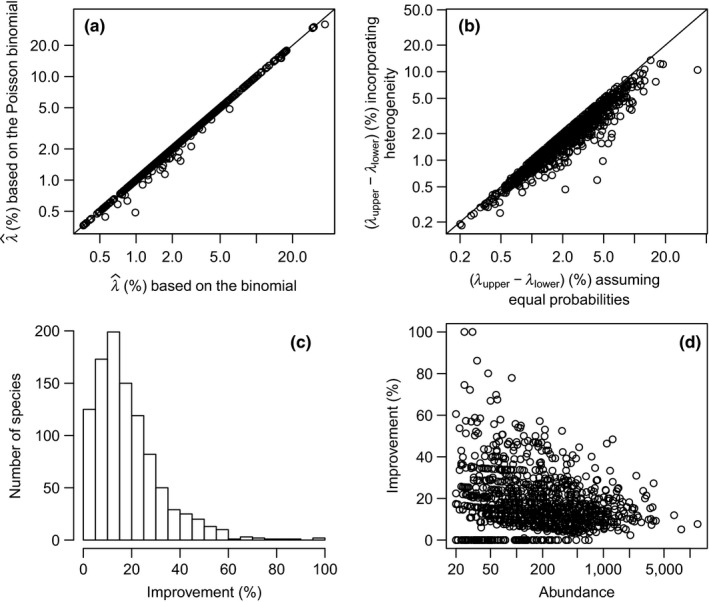
Comparison between the species‐level mortality rates obtained with the binomial distribution and constant probability of survival *p* = *k*/*n* (*x* axes in a & b) and the mortality rates obtained assuming varying probability of survival (*y* axes in a & b). (a) Comparison of central estimates, based on the most likely *k*. (b) Comparison of the width of the 95% confidence interval. Diagonals in panels a & b represent 1:1 relationships. (c) Relative improvement of using the Poisson binomial distribution (or assuming heterogeneity in the probability of survival) versus using the binomial distribution (or assuming homogeneity in the probability of survival). “Improvement” is defined as (*W*_b_ − *W*_Pb_)/*W*_b_, where *W*_b_ and *W*_Pb_ is the width of the 95% confidence interval for the binomial and the Poisson binomial distribution, respectively. (d) There was no obvious relationship between the species abundances and the relative improvement obtained by the incorporation of individual‐level variability in the probability of survival

I rescaled the vector of individual‐level probability of survival, so the mortality rates calculated with the observed number of survivors and those based on the most likely number of survivors (according to the Poisson binomial distribution) yielded almost the same results (Pearson's *r* > .99; Figure 5a). The use of varying probabilities increased the precision in the estimates of mortality rates in virtually all cases (Figure 5b), reducing the width of the confidence interval (*λ*_upper_ − *λ*_lower_) by on average ~20%. There was substantial variation in the magnitude of the improvement (Figure 5c) but it was unrelated to the species abundances (Figure 5d).

## 4 DISCUSSION

### 4.1 Take‐home message

Incorporating the estimated heterogeneity in individual‐level probability of survival provides more precise estimates of mortality rates. Any relevant source of information should be useful to estimate the vector of varying probabilities of success required, ${\hat{p}}_{1},\;{\hat{p}}_{2},\;\ldots ,\;{\hat{p}}_{n}$. Here, I have examined the (almost) worst‐case scenario, where only the position of the observations is known. The spatial dependence in mortality, as inferred from the spatial pattern of dead and surviving individuals, is sufficient to increase noticeably the precision in the estimation of species‐level mortality rates of tree species in the tropical forests of Lambir.

### 4.2 We know that the probability of survival is not constant. Yet, we use the binomial distribution

Either explicitly or implicitly, the binomial distribution assumes equal mortality rates between individuals and/or completely random spatial distribution of the different species or subgroups with different mortality rates. None of these two ecological assumptions is close to reality. In tropical forests, there are high levels of habitat preference among tree species (Baldeck et al., 2012; Davies et al., 2005; John et al., 2007; Lee, Davies, et al., 2002; Russo, Brown, Tan, & Davies, 2008). Forest species, in general, vary more than 15‐fold in their mortality rates (Condit et al., 1995; Gonzalez‐Akre et al., 2016; the present study). Besides, different species, in different habitats, show different mortality rates; typically, individuals in more fertile soils or wet areas show higher mortality rates, associated with less conservative ecological strategies (Arellano et al., 2019; Dent & Burslem, 2016; Russo et al., 2008; Zuleta, Duque, Cardenas, Muller‐Landau, & Davies, 2017).

It is clear that even small‐scale geographical variation in mortality rates is real and detectable, both in the field and statistically from many points of view. However, it is most common for ecologists to report confidence intervals for mortality rates as if they knew nothing but *k* and *n*, under the assumption of constant probability of survival either by using different analytical approximations to the binomial distribution (Condit et al., 2006, 1995; Condit, Hubbell, & Foster, 1993; Davies, 2001; Gilbert, Wright, Muller‐Landau, Kitajima, & Hernández, 2006; Itoh et al., 2012; King, Davies, & Noor, 2006; Lewis et al., 2004; Nepstad, Tohver, Ray, Moutinho, & Cardinot, 2007; Queenborough, Burslem, Garwood, & Valencia, 2007; Welden, Hewett, Hubbell, & Foster, 1991) or by bootstrapping the binary observations directly (van Breugel et al., 2011; Thomas et al., 2013). To my knowledge, only Nascimento et al. (2005) tried to incorporate dependence in observations by obtaining confidence intervals for mortality rates by bootstrapping the samples, not the individuals, in a survey of different plots in tropical forests.

### 4.3 Estimating the individual‐level probabilities of survival

The binomial distribution cannot capture any other knowledge beyond *k* and *n*, and it returns too wide confidence intervals. This is a major limitation in our understanding of patterns and processes of tropical tree mortality (e.g., Condit et al., 1995). Less conservative approaches incorporating complementary knowledge to the raw observations on tree status (dead/alive) are justified and should be incorporated into studies on tropical forest demography and ecology. Unfortunately, estimating individual‐level probabilities of survival is a nontrivial problem. The most appropriate method will be context‐dependent: it will depend on the available covariates, on the skills of the researchers, and often on the computational power available to fit models with many species and individuals. The simplest nonspatial model is a logistic regression, where the observations are dead/alive status after a given period of time and the model predicts the probability of survival given the covariates. There are several Bayesian alternatives and rapid progresses on that front (Camac et al., 2018; Iida et al., 2014; Kohyama et al., 2018; Rüger et al., 2011). Other models incorporate the space implicitly by adding covariates that inform about the influence of the neighboring individuals (e.g., Hurst, Allen, Coomes, & Duncan, 2011; Paine et al., 2012; Uriarte, Canham, Thompson, & Zimmerman, 2004). The explicit incorporation of the space (or the spatial dependence) into these models is desirable, even if only for statistical reasons. There are entire families of methods related to spatial distribution of risks and various forms of smoothing based on generalized linear mixed models, generalized additive models, autoregressive regression models (conditional or sequential), other Poisson point process models, and some other terrifying‐sounding techniques like the integrated nested Laplace approximation with stochastic partial differential equation approach.

All these approaches are designed to gain biological understanding: they require covariates (to infer about mechanisms), some prior knowledge on the system, and are far from being automatic. In general, Bayesian modeling requires considerable craftsmanship and even artistic talent. It is not for everyone. Regarding the spatial techniques in particular, none of them are commonly used in the field of plant demography and many of them are even unknown to the average ecologist. Certainly, they cannot be seen as a drop‐in alternative to simply using the binomial distribution. Furthermore, if the goal is to just provide mortality rates in a given publication (and the study is focused on something else), it would be disproportionate to fit any type of Bayesian model of tree mortality. The methods used in this study are something in between the use of the binomial distribution (using nothing but *k* and *n*) and the sophisticated modeling techniques oriented to gain biological understanding. I provide a drop‐in alternative to existing implementations, so anyone can plug survival status and coordinates into an R function and move on (“mortality_rates_using_space” function in Appendix S1). This should work in the almost worst‐case scenario, when we know nothing but the survival status and the location of the individuals. Those interested in gaining greater biological understanding can get into deeper detail by adding covariates and fitting better models. Still, the silly, mechanism‐agnostic, and purely spatial approach presented here could serve as a useful benchmark for those embarked into a modeling effort. In particular, it should be useful to determine whether certain individuals have greater/lower probability of dying relative to what can be inferred by the spatial pattern alone. Such an exercise can give rise to interesting reflections about the ecology of the studied organism and the modeling process itself.

### 4.4 The probability map should be something between a null model and an overfitted model

The development of automated or semi‐automated methods to estimate the map of probabilities based exclusively on the location of the observations can be an important line of research linking spatial ecology and plant demography. Kernel density estimation is just one of the suitable methods, but all of them would result in some sort of probability map that smooths the binary observations. The shape of the estimated probability landscape is the key “alternative world” $\hat{F}$ in the bootstrap sense; therefore, one must select meaningful smoothing parameters for its creation. Over‐smoothing is conservative. The binomial distribution is what we get if we apply a radical smoothing; in terms of Gaussian kernels, it is the same as linking completely flat kernels (*σ* ~ ∞) to all the observations. Under‐smoothing is less conservative, and therefore, over‐fitting is a risk to consider. When incorporating individual‐level probabilities of survival, the width of the confidence intervals will decline as the probability for each individual differs more and more from ½. In the case when all the individuals have ${\hat{p}}_{i}∼0$ or ${\hat{p}}_{i}∼1$, the confidence intervals will be infinitely narrow (no uncertainty in the outcome). This problem will happen if we overfit the probability map; for example, if we choose very narrow kernels (*σ* ~ 0) while considering all the *n* observations together. In such case, we would not be reflecting the reality as it is: we would be describing a specific dataset while not making useful inferences about the population. An overfitted map, with too narrow confidence intervals, would be even less useful than the case of *σ* ~ ∞ inherent to the methods based on the binomial distribution.

One general recommendation to not to overfit the map is to estimate *p_i_* for each individual *i* by excluding the observation *x_i_*, as done here. By doing so, the probability map would be always smoother than the observations themselves. That is, the predicted aggregation of dead and surviving individuals would be always somewhat lower than the observed, which clearly limits the possibility of over‐fitting the model. Furthermore, even in the case of *σ* → 0, each ${\hat{p}}_{i}$ would tend asymptotically to *k*/*n* if the observation *i* is excluded. This is because both *D*(*x_i_* = 1) and *D*(*x_i_* = 0) would be the accumulation of very small and roughly equivalent tails of the Gaussian kernels, with ~*k* tails coming from points in *U* and ~(*n* − *k*) tails coming from points in *Z*. The effect is the same in all cases when a point is far (at distance ≫ *σ*) from any other point. Rather paradoxically, if *k* = 1 the prediction for that single point in *U* would be *E*(*x_i_*)=0 with *p_i_* = 0. The same applies if *k* = *n*−1: the prediction for the only failure in the dataset would be a success with *p_i_* = 1. The same happens internally within isolated clusters of points. The researcher should take into account the consequences of using exclusively non‐*i* observations to calculate *p_i_* and whether they distort substantially the inference on *p_i_* or the expected status of each individual.

Another general recommendation is to constraint the selection of parameters using expert domain knowledge to define the relevant scales at which the spatial pattern of the observations may contain useful information. In the case presented here, I used species‐level bandwidths (*σ*) based on the observed patterns of aggregation of dead individuals or repulsion between surviving ones. In average, the species‐level *σ* chosen was ~65% shorter than the mean distance to the nearest conspecific neighbor, while there were in average eight conspecific neighbors within 3*σ* m of any given individual. Overall, these scales (*σ* around 10–20 m) seem biologically meaningful for the studied species and did not result in overfitted probability maps. Broader kernels (larger *σ*) could have been used to obtain more conservative estimates for the confidence intervals. However, it would have been meaningless when modeling a biological phenomenon to use parameters much larger than the scales at which positions and survival/death of the different stems relate to (or inform about) each other. In the case of tropical forest tree species, negative density‐dependent processes are assumed to be weak beyond 20 m from the focal individual (Bachelot & Kobe, 2013; Chanthorn, Caughlin, Dechkla, & Brockelman, 2013; Kobe & Vriesendorp, 2011; Ledo & Schnitzer, 2014; Zhu, Comita, Hubbell, & Ma, 2015), and it is difficult to think of trees affecting each other at distances larger than the typical canopy tree height (40–50 m in Lambir). Equivalent domain‐specific considerations will apply in many other scenarios.

## 5 CONCLUSION

The estimation of observation‐level probability of success, based on neighboring observations, results in a probability map. This map, combined with the Poisson binomial distribution and bootstrapping, results in proportion estimates equivalent to those obtained from the methods based on the binomial distribution. The precision of such estimates, however, increases. This is, to some degree, inevitable, since the binomial distribution is implicitly a null model uninformed about the distribution of observation‐level probability of success other than the average value, *k*/*n*. A case study calculating mortality rates of tree species in Lambir Hills National Park (Malaysia) yielded central estimates comparable to those obtained using the existing methods, with a significant reduction in the width of the 95% confidence intervals. The method described here, with modifications, should be useful to reduce uncertainty in the estimation of proportions related to any spatially structured phenomenon with two possible outcomes. In general, the Poisson binomial distribution could easily substitute the binomial distribution as the standard approach in estimating proportions, at least when studying complex phenomena for which it is known that the probability of success is not constant.

## CONFLICT OF INTEREST

None declared.

## AUTHOR CONTRIBUTION

Single‐authored paper. The Lambir data were kindly provided by collaborators ascribed to the Forest Global Earth Observatory network.

## Data Availability

The data are archived in the Smithsonian Institution's Forest Global Earth Observatory site and can be accessed by request from: http://ctfs.si.edu/Public/plotdataaccess/index.php.
